# Modeling of Structure Effect for Ferroelectric Capacitor Based on Poly(vinylidene fluoride-trifluoroethylene) Ultrathin Films

**DOI:** 10.3390/polym10010006

**Published:** 2017-12-22

**Authors:** Long Li, Xiuli Zhang, Hongzhen Chen, Xiaohui Sun, Haidong Yuan, Haisheng Xu

**Affiliations:** 1 School of Mathematics, Physics and Statistics, Shanghai University of Engineering Science, Shanghai 201620, China; m050116108@sues.edu.cn (L.L.); sunray@sues.edu.cn (X.S.); 2 School of Materials Engineering, Shanghai University of Engineering Science, Shanghai 201620, China; 3 Department of Mechanical and Automation Engineering, The Chinese University of Hong Kong, Hong Kong, China; hzchen@link.cuhk.edu.hk (H.C.); hdyuan@mae.cuhk.edu.hk (H.Y.); 4 Department of Physics, East China University of Science and Technology, Shanghai 200237, China; 5 Kunshan Hisense Electronics Co., Ltd., Kunshan 215300, China

**Keywords:** P(VDF-TrFE), polarization, depolarization field, fatigue

## Abstract

The characteristics of ferroelectric capacitors with poly(vinylidene fluoride-trifluoroethlene) (P(VDF-TrFE)) films have been studied at different structures of cell electrodes. It is suggested that the effect of electrode structures could induce changes of performance. Remarkably, cells with line electrodes display a better polarization and fatigue resistance than those with flat electrodes. For P(VDF-TrFE) ultrathin films with different electrode structures, the models of charge compensation mechanism for depolarization field and domain fatigue decomposition are used to explain the effect of electrode structure. Furthermore, the driving voltage based on normal speed-functionality is designed, and the testing results show that the line electrode structure could induce a robust switching, which is determined by the free charges concentration in active layer. These findings provide an effective route to design the optimum structure for a ferroelectric capacitor based on P(VDF-TrFE) copolymer ultrathin film.

## 1. Introduction

With the rapid development of intelligent electronics industry, ferroelectric materials have attracted a great deal of attention as a kind of functional material. Compared with inorganic ferroelectric oxides materials, organic ferroelectric polymers have some outstanding advantages, such as low-voltage operation [1,2,3,4,5], high flexibility [2,6,7,8], low cost [2,3,9,10], and so on. Poly(vinylidene fluoride) (PVDF) and its copolymer poly(vinylidene fluoride-trifluoroethylene) P(VDF-TrFE), at present, are the most studied organic ferroelectric materials due to relatively excellent ferroelectricity [10,11], piezoelectricity [12,13,14], and dielectric [15,16]. It is well known that P(VDF-TrFE) exhibits much better ferroelectric properties and stability of structure than PVDF in polarized state [17,18]. As a result, P(VDF-TrFE) is considered as one of the most promising candidates for applications of organic electronic devices, such as sensors [1,10,19,20,21], actuators [1,19,20,21,22], and non-volatile memories [9,23,24,25]. Particularly for flexible electronic applications, controlled and predictable reversal characteristics are required. P(VDF-TrFE) copolymers have yielded promising results in terms of capacitor behavior. By combining organic ferroelectric and semiconductor materials, nonvolatile memory can be attained [26]. However, the structure of the capacitor in the ferroelectric film is known to be of critical importance in device optimization [24].

In most cases, P(VDF-TrFE) is applied in electronic devices in the form of a film. It is widely accepted that the performance of P(VDF-TrFE) film deteriorates with the decrease of the thickness of the film, especially for the film with thickness below 100 nm [27,28,29]. However, to maintain a suitable capacitance, a demand for ultrathin ferroelectric films for device miniaturization has arisen. Therefore, it is crucial how to improve the performance of these organic devices with ultrathin films in practical application. Through the efforts of many researchers, several means have been proposed to reduce the deterioration caused by the structural dimension in ultrathin ferroelectric films, such as suitable annealing temperature [10,18,30], cooling rate [31], electric field [32], VDF content [18,32], and so on. Besides the means mentioned above, optimizing the structure of capacitors is also an important method to improve the performance of P(VDF-TrFE) films. A half-micron ferroelectric memory cell technique with stacked capacitor structure has been reported in the application of non-volatile memories [33]. Moreover, ferroelectric capacitor devices with line or circular structure electrodes were reported by different researchers [1,34,35]. Our previous research also reported that the sandwiched structure using electroactive polymers as an interlayer between electrodes shows many excellent properties [36]. Although many significantly improved performances have been achieved, the physical mechanism and the model of such a structure effect of P(VDF-TrFE) films in a suitable capacitance are unclear. Therefore, it is necessary to investigate the effect of structures in ferroelectric polymers. This work mainly focuses on the analysis on the electrode structure effect on the performance of capacitors. It is noteworthy that the supercapacitors with high performance in energy and power density develop rapidly recently due to energy demand [37]. On the other, the polarization behavior of the optimized capacitor is one of the important issues for ferroelectric thin films in view of memory applications, which determines how fast such devices can operate.

In this work, the universal and easy-to-implement models for ferroelectric capacitors with line or flat electrode are studied. Concentrating on the switching in ferroelectric capacitors, these models can describe the dynamical process of the polarization and switching behavior. In order to systematically discuss the effect of different electrode structures (e.g., flat and line electrode) on capacitor properties, capacitors with different types of electrode structures were fabricated. We found that the cells with line electrodes display better polarization and fatigue resistance than those with flat electrodes.

## 2. Materials and Methods

**Figure 1:**
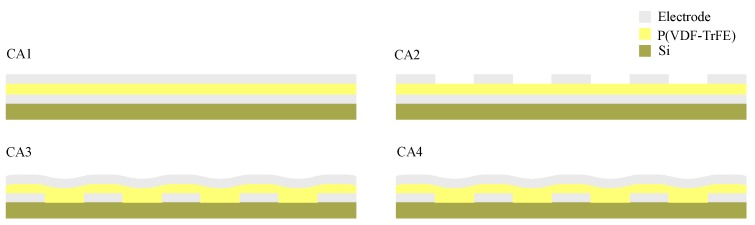
The scheme depiction of capacitor with different electrode structures. (CA1) Flat-flat capacitor with flat top and bottom electrode structure. (CA2) Line-flat capacitor with line top and flat bottom electrode structure. (CA3) Flat-line capacitor with flat top and line bottom electrode structure. (CA4) Line-line capacitor with line top and bottom electrodes perpendicular to each other.

For metal-ferroelectric-metal capacitor devices with P(VDF-TrFE) ultrathin films, we used a set of four model cells where the effects of different structure patterning could be studied systematically. These cells are called a Crosspoint Array electrode (CA1–4) and have the following combinations of flat and line electrodes, as shown in Figure 1. The top and bottom metal electrodes are fabricated in two different ways: (1) For the top flat electrode, Ti (60 nm) is directly deposited on the P(VDF-TrFE) surface, while for the bottom flat electrode, the silicon wafer is covered globally with Ti; (2) A line electrode can be used as the top or bottom electrode, which is evaporated with a shadow mask, and the width and spacing of the line electrodes are both 250 nm. The thickness of the ferroelectric polymer films of CA1, CA2 is 76 nm, and that of CA3, CA4 is both 57 nm, where the thickness of the film is the distance between the bottom and top electrodes.

The ultrathin films of P(VDF-TrFE) copolymer with mole ratio of VDF-TrFE 70/30 were deposited on silicon wafer by a spin-coating technique from a 2 wt % diethyl carbonate solution at 25 °C. The P(VDF-TrFE) copolymer was provided by Kunshan Hisense Electronic Co., Ltd. (Suzhou, China). The film thickness was measured by Alpha-step 500 surface profiler. The bottom electrodes were fabricated on a 50 nm-thick SiO_2_ substrate. The P(VDF-TrFE) solution was filtered in advance using 0.1-μm filters. The operating temperature and frequency were 25 °C and 10 Hz, respectively. The samples were then annealed at 130 °C for 1 h. The ferroelectric measurements were obtained on Precision Pro Ferroelectric tester manufactured by Radiant Technologies (Albuquerque, NM, USA).

**Figure 2:**
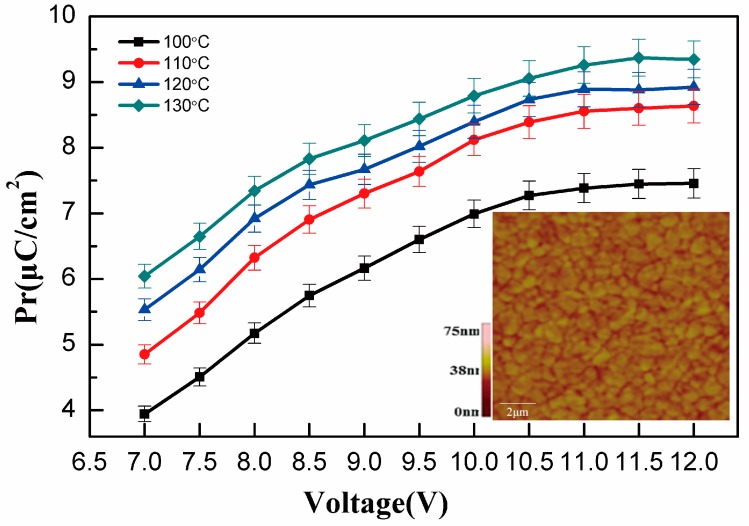
The *P_r_* as a function of driver voltage from 7 V to 12 V at different anneal temperatures (100–130 °C). The inset shows CA2 sample AFM (Atomic Force Microscopy) images of a 76-nm P(VDF-TrFE) film.

Figure 2 shows that the polarization increases with the voltage. For the sample of 130 °C annealing, it exhibits a polarization of 9.3 μC/cm^2^. The polarization enhancement was observed previously, where it might be due to anneal-affected crystallization. In all our reports [29] there is an apparent correlation between the polarization enhancement. The inset shows the CA2 sample AFM (Atomic Force Microscopy) image of a 76-nm P(VDF-TrFE) film spin-coated from DEC (Diethyl Carbonate) solutions. From the inset, it can be seen that the surface is flatter, and grains with a size of 1~2 μm are shown. Worm-like grains are observed and the crystallite regions are assumed to be in the form of lamellae, as is common in polymers.

## 3. Results and Discussion

### 3.1. Polarization 

**Figure 3:**
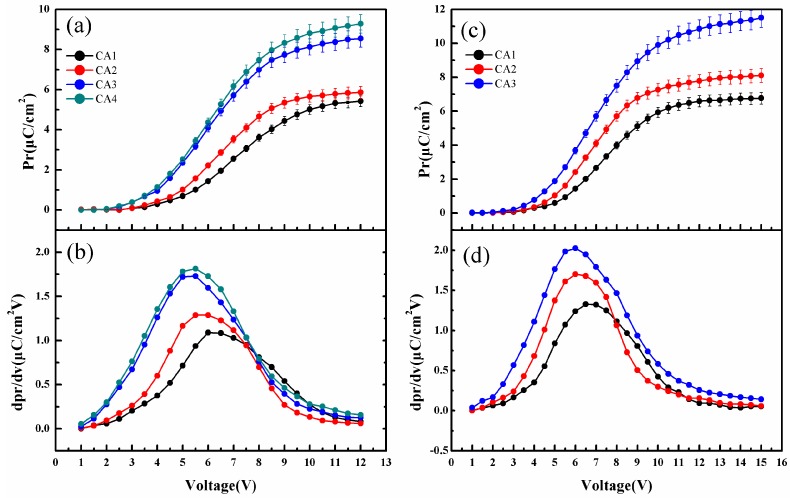
*Pr* and the first derivative value of *P_r_*. (**a**) *P_r_* as a function of the driver voltage from 1 V to 12 V; (**b**) The first derivative value of *P_r_* with the driver voltage from 1 V to 12 V; (**c**) *P_r_* as a function of the driver voltage from 1 V to 15 V; (**d**) The first derivative value of *P_r_* with the driver voltage from 1 V to 15 V.

Figure 3a,c show the value of *P_r_* (remanent polarization) measured from drive voltage on the films from 1 V to 12 V and 1 V to 15 V, respectively. A typical “S” shaped curve due to ferroelectric properties is observed for all samples [38]. The first derivative value of *P_r_* as a function of voltage is shown in Figure 3b,d. The samples of CA3 and CA4 with 250-nm spacing line bottom electrodes have the highest value of *P_r_*, and the maximum values of *P_r_* are 8.5 μC/cm^2^, 9.3 μC/cm^2^, respectively. The first derivative extreme value of *P_r_* gives the distinct voltage at 5 V and 6 V. On the other hand, the samples of CA1 and CA2 with normal flat bottom electrodes are known for relatively low polarization. However, for the line top electrode of CA2, the polarization performance is improved compared to the flat top electrode of CA1. The maximum *P_r_* values of CA1 and CA2 are 5.4 μC/cm^2^ and 5.8 μC/cm^2^, respectively. From these traits, the polarization difference can be attributed to two aspects: the block layer structure effect and the line bottom electrode structure effect.

**Figure 4:**
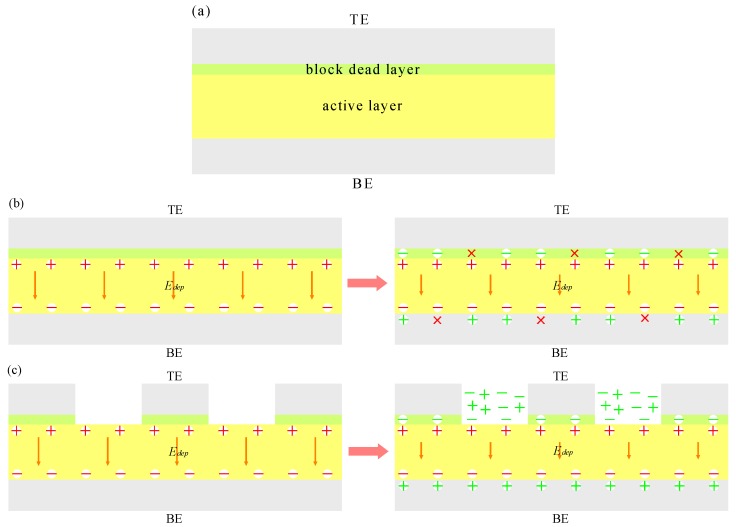
(**a**) Model of the ferroelectric capacitor with a P(VDF-TrFE) film including an active layer and a block dead layer. The top and bottom electrodes are indicated by TE and BE, respectively. (**b**) The model of charge compensation mechanism with a flat electrode for the depolarization field. (**c**) The model of charge compensation mechanism with a line electrode for the depolarization field.

Figure 4a gives a model of this ferroelectric capacitor, which includes an active layer and a block dead layer. During the top metal electrode evaporation process, the chemical reaction between the Ti electrode and the polymer film results in the formation of a block dead layer. In principle, there could be such a block dead layer between the electrode and the P(VDF-TrFE) film. Many references show the presence of a block dead layer in addition to the active layer in the ferroelectric film capacitor structure [36,39,40,41]. Our previous XPS (X-ray photoelectron spectroscopy) results also clearly revealed the formation of TiFx during the top electrode deposition [27]. It is generally accepted that the polarization in an active layer originates from electric dipoles orientation, which gives rise to a depolarization field (*E_dep_*). However, *E_dep_* in ferroelectric capacitors can cause a marked effect on polarization values. The design of electronic devices using ferroelectric film capacitors should take into account the effects of *E_dep_*. C. T. Black [42] used a ferroelectric capacitor in series with a linear capacitance to simulate the effect of *E_dep_* on polarization. The value of *E_dep_* is adjusted by changing the amount of compensating charge, which is presented in the following equation:

(1) $$P_{r}=P_{r}^{*}\cdot \frac{1}{1+\delta }$$

where P_r_* is the maximum value of the ferroelectric polarization when complete charge compensation (*δ* = 0) occurs in the ferroelectric capacitor. Here *δ* is used to characterize the effect of *E_dep_*. A large *δ* means that *E_dep_* causes significant suppression of the polarization. Without any compensating charge, *δ* = 4.7 and polarization will be reduced to roughly 18% of the maximum value [42]. Therefore, to stabilize the polarization, one needs to supply compensation charges at the surfaces of the material, because compensation charges weaken the inhibitory effect of the depolarization field [24,43].

In Figure 4b,c, the models of the charge compensation mechanism for the depolarization field are proposed. It can be seen that the block dead layer and free charges are both in existence in these models. The electrons originating from the electrodes nearby could supply the amount of charges needed for the compensation of the ferroelectric dipoles to stabilize the domain during the switching process [36]. However, the different *δ* is obtained due to the difference of electrode structures. As shown in Figure 4b, for the cells without line electrodes, the ferroelectric active layer is covered by a block dead layer with a large area. What is more, a small number of free charges exist in the ferroelectric thin film surface, and can hardly get through the block dead layer. Therefore, the depolarization field in an active layer cannot be completely compensated by the free charges, which leads to a relatively high *δ*_1_. A schematic depiction of the charge compensation mechanism with a line electrode for the depolarization field is presented in Figure 4c. The contact area between the line electrode and polymer is much smaller than that of the flat electrode and polymer owing to the effect of line electrodes spacing, indicating that the ferroelectric active layer is covered by a block dead layer with a relatively small area. A large number of free charges exist in the ferroelectric thin film surface, and can easily get through the block dead layer. Thus, the depolarization field in an active layer is compensated more by a large number of free charges, which leads to a low *δ*_2_. The inhibition effect of the depolarization field to ferroelectric thin film polarization is decreased. When *δ*_1_ > *δ*_2_, *P_r_*_1_ (without a line electrode) is smaller than *P_r_*_2_ (with a line electrode). Therefore, the polarization property of ferroelectric thin films with a line electrode is improved.

On the other hand, compared to the effect of the block dead layer, the effect of the line bottom electrode structure is more interesting for the polarization. As shown in Figure 3a, the cells (CA3, CA4) with line bottom electrodes exhibit better polarization than those (CA1, CA2) with normal flat bottom electrodes, which may be attributed to the effect of the line bottom electrode structure. To obtain further insights on the effect of the line bottom electrode structure, the cells with different electrode step heights were prepared (see Section 3.4). However, it is indicated that the effect of the line bottom electrode structure is independent on electrode step heights, and further line bottom electrode mechanistic studies as well as the development of related processes are ongoing in our laboratory.

### 3.2. Effect of Electrode Structure on Fatigue

Table 1 shows the *P_r_* before (BF) and after (AF) fatigue test with different electrode structures, before and after 10^6^ switching cycles. It is clear that the electrode structure effect exists in this system. For the sample of CA4 with line top and bottom electrodes, the *P_r_* is as high as 6.90 μC/cm^2^ and the value of the fatigue ratio (AF/BF) is at a high level of 0.79 under 10^6^ switching cycles. Therefore, the sample of CA3 with flat top and line bottom electrodes maintains the same *P_r_* value as CA4, but a worse fatigue ratio. However, the value of *P_r_* (4.65 μC/cm^2^) and the ratio (AF/BF) (0.44) of the CA1 sample with flat top and bottom electrodes are lower than those of the CA4 sample (6.90 μC/cm^2^, 0.79), with line top and bottom electrodes. Correspondingly, for CA2, which is prepared with line top and flat bottom electrodes. The *P_r_* and ratio (AF/BF) also decrease to 5.02 μC/cm^2^ and 0.67, respectively, 

Thereby, the effect of electrode structure is clearly demonstrated based on different CA cell models. Firstly, the line structure can improve ferroelectric performance due to the charge compensation mechanism in the activation layer. Our previous reports showed that the influence of compensating free charge is one of important factors to improve ferroelectric fatigue [27]. The line electrodes tend to supply more compensating charges between the polymer and electrode layer. This is in accordance with the results shown in Figure 3. Secondly, as the line electrode is prepared as a top/bottom electrode (CA2, CA3), better performance (polarization or fatigue) could be achieved in comparison to the cell with only flat electrodes (CA1). Finally, to further demonstrate the superiority of the line electrode structure, we used line electrodes as bottom and top electrodes. The *P_r_* and ratio (AF/BF) of Table 1 show that in all the fatigued capacitor test results, the sample of CA4 (with top and bottom line electrodes) exhibited excellent ferroelectric performance and fatigue resistance.

**Figure 5:**
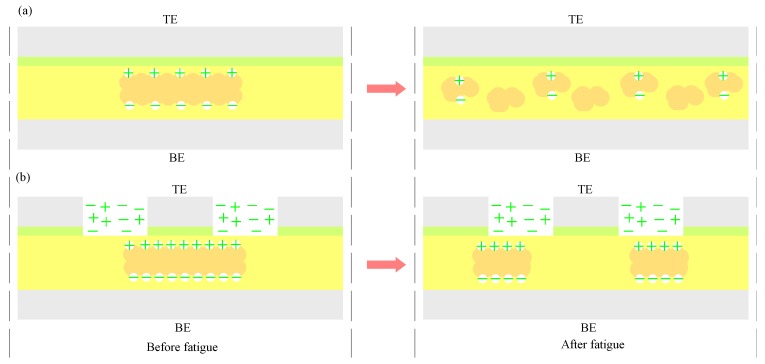
Schematic of polarization reversal fatigue progress. (**a**) Schematic of domain degradation progress without enough free charges; (**b**) Schematic of domain degradation progress with enough free charges.

As is well-known, the fatigue of the domain occurs in the process of ferroelectric reversal. To understand the mechanism of the ferroelectric polymer domain fatigue, the schematic depiction of domain fatigue is presented in Figure 5. Fatigue in organic ferroelectric materials has been ascribed to charge trapping. Injected charges become trapped at crystalline boundaries and defects, thereby locking the domain walls and reducing the polarization [44]. Here we systematically describe the process of fatigue from the viewpoint of domain degradation. The models start with the assumption that only in an ideal case is the original state homogeneous and in a single domain. As shown in Figure 5a, the schematic of domain deterioration with the flat electrode structure is displayed. It can be seen that the ferroelectric domain is a large-volume single domain and has strong activity before fatigue, after more than 1 × 10^6^ of switching cycles; however, the ferroelectric domain is decomposed from a single domain into many small volumes and the balance of the switching domain is destroyed. On the other hand, the activity of the ferroelectric domain also decreases. In comparison to the flat electrode structure, the deterioration of the ferroelectric domain also occurs in line electrode structure P(VDF-TrFE) film capacitors. However, this deterioration can be weakened by the use of the line electrode structure, as shown in Figure 5b, where it can be seen that a large number of available free charges exist in the P(VDF-TrFE) film active layer surface or interior. With enough charge concentration, ferroelectric domains are usually surrounded by the free charges in the switching progress, which often keep domains alive, especially to maintain domain balance. Therefore, the deterioration and activity reduction of domain are both improved in the switching process. The cell with the line electrode structure exhibited higher remnant polarization and an enhancement of fatigue resistance compared to the other without the line electrode structure, which may be attributed to line electrode effect.

### 3.3. Effect of Electrode Structure on Driving Voltage

The choice of driving voltage (*V_d_*) is also one of the important factors for the performance of ferroelectric thin films. The device will not work properly if the voltage applied is too small, and may break if the voltage is too large. For example, as shown in Figure 3c, with the increase of voltage, CA4 is broken by the 15-V driving voltage. Therefore, the driving voltage data of CA4 is lacking in Figure 3c, indicating that it is necessary to choose the right voltage. The driving voltage data of cells with different electrode structures are presented in Table 2, which is based on normal speed-functionality drive choice. From the table, it can be seen that in all test results, CA1 with flat top and bottom electrodes requires the highest driving voltage (10.1 V). On the contrary, the lowest *V_d_* (9.3 V) is observed in CA4, with line top and bottom electrodes. In addition, the driving voltage of CA2 and CA3 are 9.8 V and 9.6 V, respectively.

Compared to CA1, the driving voltage of CA2 and CA3 with a line top or bottom electrode decreases by 0.3 V and 0.5 V, respectively. What is more, the driving voltage of CA4 reduces by 0.8 V. This demonstrates, once more, that the electrode structure effects are real in this system. On the basis of the analysis in Section 3.1, owing to the electrode structure effects, the cells with line electrodes (top or bottom) have satisfactory ability to resist fatigue and depolarization field. The larger the depolarization field, the greater the driving voltage required. Therefore, a relatively small voltage (9.3 V) is required for CA4, and a relatively big voltage (10.1 V) is needed for CA1.

### 3.4. Polarization Independence of the Electrode Step Heights

**Figure 6:**
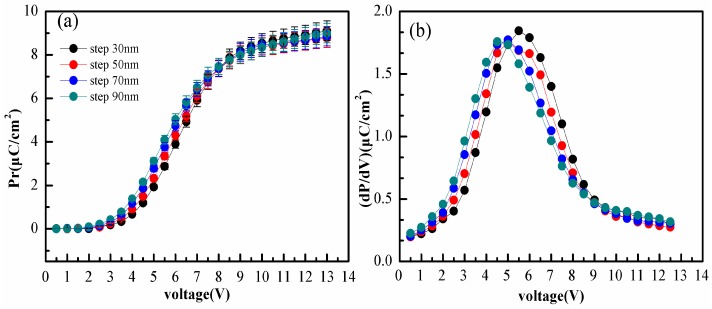
*P_r_* and the first derivative value of *P_r_*. (**a**) *P_r_* as a function of the driver voltage for the films with varying step heights; (**b**) The first derivative value as a function of voltage for the films with varying step heights.

In order to further investigate the effect of the line bottom electrode structure, cells with different electrode step heights were prepared. *P_r_* and the first derivative value of *P_r_* as a function of voltage are displayed in Figure 6. As can be seen in Figure 6a, one salient feature of the data is that the *P_r_* curves still show the typical “S” shape, in accordance with Figure 3a. What is more interesting is the result of *P_r_*. Although the cells have different electrode step heights, very similar *P_r_* values are obtained. This suggests that the effect of the line bottom electrode structure is independent of electrode step heights. On the other hand, as shown in Figure 6b, the electrode step heights are 30 nm, 50 nm, 70 nm, and 90 nm, and the voltage of the first derivative extreme values are 6 V, 6 V, 5 V, and 5 V, respectively. The different voltage values are recorded at extremes due to the diversity of the electrode step heights.

## 4. Conclusions

In summary, we observed a critical dependence of the properties of P(VDF-TrFE) ferroelectric films on the electrode structure. The difference of ferroelectricity could be induced by the line and flat electrode structures. Furthermore, we investigated the cells with line electrodes and observed better polarization and fatigue resistance in comparison to cells with flat electrodes. The analyses presented indicate that the electrode structure effects, including the effect of the block dead layer and line bottom electrode structure effect, are mainly responsible for the performance of the ferroelectric in this system. The switching characteristics are strongly affected by electrode structure factors and the model of free charge compensation mechanism is proposed to explain this effect. The free charges have influence on the depolarization field as well as the domain balance, which is induced by the electrode structure effect. Furthermore, the optimum driving voltage of ferroelectric capacitors with different electrode structures are obtained. These findings provide an effective way to achieve the excellent performance of P(VDF-TrFE) ferroelectric film capacitors.

## Figures and Tables

**Table 1:** *Pr* before (BF) and after (AF) fatigue test with different electrode structures.

Cell name	CA1	CA2	CA3	CA4
Top electrode	Flat	Line	Flat	Line
Bottom electrode	Flat	Flat	Line	Line
*P_r_*(BF) (μC/cm^2^)	4.65	5.02	6.93	6.90
*P_r_*(AF) (μC/cm^2^)	2.98	3.40	3.80	5.43
Ratio (AF/BF)	0.44	0.67	0.56	0.79

**Table 2:** Driving voltage test result with different electrode structures.

Cell name	CA1	CA2	CA3	CA4
*V_d_*	10.1 V	9.8 V	9.6 V	9.3 V
